# Liver fibrosis and accelerated immune dysfunction (immunosenescence) among HIV-infected Russians with heavy alcohol consumption - an observational cross-sectional study

**DOI:** 10.1186/s12876-019-1136-4

**Published:** 2019-12-31

**Authors:** Kaku So-Armah, Matthew Freiberg, Debbie Cheng, Joseph K. Lim, Natalia Gnatienko, Gregory Patts, Margaret Doyle, Daniel Fuster, Dmitry Lioznov, Evgeny Krupitsky, Jeffrey Samet

**Affiliations:** 1 Department of Medicine, Boston University School of Medicine, 801 Massachusetts Avenue, Crosstown Building, 2nd Floor, Boston, MA 02118 USA; 2 Department of Medicine, Vanderbilt University School of Medicine; Nashville Veterans Affairs Medical Center, Tennessee Valley Healthcare System, Nashville, TN USA; 3 Department of Biostatistics, Boston University School of Public Health, Boston, MA USA; 4 Department of Internal Medicine, Yale University School of Medicine, New Haven, CT USA; 5 Department of Medicine, Boston Medical Center, Boston, MA USA; 6 Department of Biostatistics and Data Coordinating Center, Boston University School of Public Health, Boston, MA USA; 7 Department of Pathology and Laboratory Medicine, University of Vermont, Burlington, VT USA; 8 Department of Internal Medicine, Hospital Universitari Germans Trias i Pujol, Universitat Autònoma de Barcelona, Badalona, Spain; 9 Department of Infectious Diseases and Epidemiology, Smorodintsev Research Institute of Influenza, First Pavlov State Medical University, St. Petersburg, Russia; 10 First Pavlov State Medical University, V. M. Bekhterev National Research Medical Center for Psychiatry and Neurology, St. Petersburg, Russia; 11 Department of Medicine, Boston Medical Center, Boston University School of Medicine, Boston, MA USA

**Keywords:** HIV, Liver fibrosis, Immune senescence, Russia, Alcohol

## Abstract

**Background:**

The multifactorial mechanisms driving negative health outcomes among risky drinkers with HIV may include immunosenescence. Immunosenescence, aging of the immune system, may be accentuated in HIV and leads to poor outcomes. The liver regulates innate immunity and adaptive immune tolerance. HIV-infected people have high prevalence of liver-related comorbidities. We hypothesize that advanced liver fibrosis/cirrhosis is associated with alterations in T-cell subsets consistent with immunosenescence.

**Methods:**

ART-naïve people with HIV with a recent history of heavy drinking were recruited into a clinical trial of zinc supplementation. Flow cytometry was used to characterize T-cell subsets. The two primary dependent variables were CD8+ and CD4+ T-cells expressing CD28-CD57+ (senescent cell phenotype). Secondary dependent variables were CD8+ and CD4+ T-cells expressing CD45RO + CD45RA- (memory phenotype), CD45RO-CD45RA+ (naïve phenotype), and the naïve phenotype to memory phenotype T-cell ratio (lower ratios associated with immunosenescence). Advanced liver fibrosis/cirrhosis was defined as FIB-4 > 3.25, APRI≥1.5, or Fibroscan measurement ≥10.5 kPa. Analyses were conducted using multiple linear regression adjusted for potential confounders.

**Results:**

Mean age was 34 years; 25% female; 88% hepatitis C. Those with advanced liver fibrosis/cirrhosis (*N* = 25) had higher HIV-1 RNA and more hepatitis C. Advanced liver fibrosis/cirrhosis was not significantly associated with primary or secondary outcomes in adjusted analyses.

**Conclusions:**

Advanced liver fibrosis/cirrhosis was not significantly associated with these senescent T-cell phenotypes in this exploratory study of recent drinkers with HIV. Future studies should assess whether liver fibrosis among those with HIV viral suppression and more advanced, longstanding liver disease is associated with changes in these and other potentially senescent T-cell subsets.

## Background

Heavy alcohol use is more prevalent among people living with human immunodeficiency virus/acquired immunodeficiency syndrome (PLWHA) than among uninfected people [1] and is associated with liver disease and multiple negative health outcomes [2]. Alcohol related liver injury among PLWHA is compounded by high prevalence of liver-related comorbidities occurring among PLWHA such as viral hepatitis. The multifactorial mechanisms driving negative health outcomes among PLWHA who are risky drinkers are still being elucidated [3–7].

The liver plays a critical role in metabolic detoxification and immune regulation [8]. It receives blood from the hepatic arteries and the portal venous system. Blood from the portal venous system contains nutrients, metabolic products as well as toxins and antigens. Thus, the liver must balance immune activation from antigen exposure with preventing damage to hepatocytes and surrounding tissues from the antigenic response.

We hypothesize that fibrotic or cirrhotic livers of PLWHA have diminished capability to maintain this balance, resulting in chronic immune activation and ultimately, premature immune exhaustion (immunosenescence). The objective of this study is therefore to explore the association between advanced liver fibrosis/cirrhosis and alterations in T-cell subsets among ART-naïve HIV-infected Russians with heavy alcohol consumption.

## Methods

### Participants

Study participants were from the Russia ARCH (Alcohol Research Collaboration on HIV/AIDS) and ZINC HIV (Zinc for INflammation and Chronic disease in HIV) studies. Russia ARCH is a longitudinal cohort of PLWHA with varying levels of alcohol consumption. ZINC HIV is a randomized double-blinded placebo controlled clinical trial (ClinicalTrials.gov identifier: NCT01614626). The aim of ZINC HIV is to determine the efficacy of long-term zinc supplementation compared to placebo among PLWHA on outcomes related to mortality risk, cardiovascular disease risk, microbial translocation, inflammation and HIV disease progression [9]. Participants in the ZINC HIV trial were antiretroviral therapy naïve at enrollment and reported heavy drinking within the 30 days prior to enrollment. Heavy drinking was defined according to National Institutes on Alcohol Abuse and Alcoholism (NIAAA) risky drinking criteria as > 4 standard drinks in a day (or > 14 standard drinks/week) for men and > 3/ day (or > 7/week) for women. The first 250 participants in Russia ARCH had T-cell phenotyping by flow cytometry. All ZINC participants had liver enzymes and platelets measured and Fibroscan imaging performed. The current study focuses on the overlapping subset of participants in both Russia ARCH and ZINC who had available liver fibrosis data and T-cell phenotypes at baseline (*N* = 130).

### Main independent variable

The main exposure variable was advanced liver fibrosis/cirrhosis defined as having any of the following: FIB-4 score > 3.25, APRI score ≥ 1.5 or Fibroscan result ≥10.5 kPa [10, 11]. The FIB-4 score is calculated as the product of age (years) and aspartate aminotransferase (AST, U/L) divided by the product of platelet count (10^9^/L) and the square root of alanine aminotransferase (ALT, U/L). The resulting score was categorized as ≤3.25 (no advanced fibrosis/cirrhosis) or > 3.25 (advanced fibrosis/cirrhosis). The APRI score is calculated as AST divided by platelet count and categorized such that scores greater than or equal to 1.5 reflect advanced fibrosis/cirrhosis. By design in the ZINC HIV trial, participants with FIB-4 in the indeterminate range (i.e., 1.45 to 3.25) received Fibroscan imaging. Fibroscan enables ultrasound measurement of liver stiffness, which correlates with liver fibrosis. These data were used to confirm adequate separation of liver fibrosis exposure groups among the subset of participants on whom Fibroscan imaging was done.

### Outcome variable

We analyzed CD8+ and CD4+ T-cell phenotypes consistent with immunosenescence, the aging related decline of adaptive immune function. Immunosenescence is characterized by accumulation of CD28-CD57+ T-cells, decrease in naïve lymphocytes, and increase in memory lymphocytes that are oligoclonally expanded, resistant to apoptosis and functionally incompetent [12, 13]. The two primary outcomes were the percentage of CD8+ and CD4+ T-cells expressing the CD28-CD57+ phenotype. T-cells that lose CD28 (CD28-) or gain CD57 (CD57+) expression have undergone chronic antigenic stimulation, multiple rounds of antigen-specific replication, and have decreased ability to replicate further. These T-cell subsets are cytotoxic or immunosuppressive, and poorly regulated [14]. The CD28-CD57+ phenotype is associated with alcoholism [15], intracellular infections like HIV, [16, 17] and diseases of aging like cancer [14]. Six secondary outcomes were evaluated: CD8+ and CD4+ T-cells expressing CD45RO + CD45RA- (memory phenotype), CD45RO-CD45RA+ (naïve phenotype), or naïve:memory T-cell ratio (lower ratios associated with immunosenescence). Skewed outcomes were natural log-transformed to better approximate a normal distribution.

Flow cytometry and T-cell quantification have been previously described [18]. Briefly, heparin anticoagulated whole blood was processed within 4 h of collection at the St. Petersburg Pasteur Institute Central Clinical Diagnostic Laboratory. All reagents were from BD Biosciences. Whole blood was labeled with FITC anti-CD8, PE anti-CD45RO, PeCy5 anti-CD45RA and APC-H7 anti-CD4, or FITC anti-CD8, PE anti-CD57, PeCy5 anti-CD28 and APC-H7 anti CD4 or appropriate isotypes. Samples were incubated, red blood cells were lysed, cells were washed, and samples were fixed in paraformaldehyde. Flow cytometry was performed on a BD FACS Cantos that has been calibrated daily and compensation was set using single color controls. Flow data was analyzed with BD FACS DIVA software. Lymphocytes were gated based on their forward and side scatter. CD4+ and CD8+ cells are reported as a percent of gated lymphocytes. All other subsets are a percent of either CD4+ or CD8+ cells.

### Covariates

This study used covariate data obtained from the following instruments: 30 Day Timeline Follow Back for alcohol use, [19] Fagerström Test for Nicotine Dependence, [20] survey of co-morbidities adapted from the Veterans Aging Cohort Study patient questionnaire [21] and HIV Risk Behavior Survey (RBS) [22]. Past 30-day heavy drinking was based on Timeline Followback for alcohol use and categorized per NIAAA risky drinking criteria. HIV RNA was quantified using a polymerase chain reaction-based diagnostic (AmpliSens® HIVMonitor-FRT, Amplisens, Moscow, Russia). Hepatitis C status was based on an antibody test. Past 30-day injection drug use, current smoking and prior exposure to potentially chronic infections (hepatitis B, tuberculosis, herpes zoster) were based on self-report. Covariates were potential confounders selected a priori based on the literature and clinical knowledge.

### Statistical analysis

For descriptive purposes, we compared participant characteristics at baseline by FIB-4 category (no/moderate fibrosis vs. advanced fibrosis/cirrhosis) using t-tests, Wilcoxon rank sum, chi-square or Fisher’s exact tests as appropriate. Primary and secondary outcomes were compared between the FIB-4 categories of interest using multiple linear regression adjusted for potential confounders. Regression models were adjusted for age, sex, HIV-1 RNA, injection drug use in the prior 30 days, current smoking, time since HIV diagnosis, hepatitis C, and exposure to chronic infections (hepatitis B, tuberculosis, herpes zoster). CD8+ and CD4+ naïve to memory ratios and CD4 + CD28-CD57+ T-cell subsets were natural log-transformed to improve normality and back-transformed for ease of interpretation. For back transformed T-cell subsets, measures of effect are reported as a ratio of means for comparisons between those with advanced fibrosis/cirrhosis compared to those without. Confirmatory analyses were performed using median regression models, which are more robust to departures from normality and the presence of outliers than linear regression models [23, 24]. Additional exploratory analyses were conducted evaluating FIB-4 as a continuous variable. As an initial step, generalized additive models (GAMs) were used to assess the form of the relationship between FIB-4 and outcomes and whether the linearity assumption was appropriate. Based on the GAMs, linear or piecewise linear regression models were then used to evaluate whether FIB-4 was associated with CD4 + CD28-CD57+ and CD8 + CD28-CD57+ T-cell subsets. For example, in the analyses of the association between FIB-4 and CD8 + CD28-CD57+ T-cells, the GAM suggested separate slopes at a FIB-4 value of approximately 2.7. We therefore subsequently fit a piecewise linear regression model estimating separate slopes for those with FIB-4 less than or equal to 2.7 versus those above this threshold and tested each slope separately. Analyses were performed with SAS 9.3 statistical software. The datasets used and/or analyzed during the current study are available from the corresponding author on reasonable request.

## Results

Among the 130 participants included in the current study, 25 (19%) were categorized as having evidence of advanced liver fibrosis/cirrhosis (Table 1). The participants were all Caucasian, predominantly younger men (mean age 34 years) with high prevalence of heavy drinking (90%), hepatitis C antibody positivity (85%) and mean time since HIV diagnosis of 7 years. Mean log_10_ HIV-1 RNA copies/mL was 4.5. Those with advanced liver fibrosis/cirrhosis had more prevalent hepatitis C co-infection. Among those with Fibroscan data, median Fibroscan scores were higher among those with evidence of liver fibrosis compared to those without (8.0 vs. 6.2) (*p* < 0.05 for comparisons by liver fibrosis category).

**Table 1:** Baseline characteristics of study population by liver fibrosis status among ART-naïve people living with HIV/AIDS (PLWHA) with recent heavy alcohol use

	Overall	No advanced fibrosis/cirrhosis	Advanced fibrosis/cirrhosis	*p*-value
N	130	105	25	–
Mean age (SD)/years	33.5 (6.0)	33.6 (6.2)	33.1 (4.9)	0.74
Female sex	33 (25%)	26 (25%)	7 (28%)	0.74
Heavy drinking^a^	118 (91%)	95 (90%)	23 (92%)	0.81
Injected drugs use (past 30 days)	46 (35%)	38 (36%)	8 (32%)	0.69
Current regular smoker	111 (85%)	87 (83%)	24 (96%)	0.09
Self-reported history of hepatitis B	43 (33%)	34 (32%)	9 (36%)	0.73
Self-reported history of herpes zoster	28 (22%)	20 (19%)	8 (32%)	0.16
Self-reported history of tuberculosis	16 (12%)	11 (11%)	5 (20%)	0.19
Hepatitis C antibody positive	114 (88%)	89 (85%)	25 (100%)	0.04
Mean years since first positive HIV test (SD)	6.8 (5.0)	6.7 (4.9)	7.3 (5.4)	0.54
Mean platelet count (SD)	211 (74)	226 (65)	148 (75)	< 0.01
Mean ALT (SD)/Units per liter	52 (74)	38 (40)	109 (136)	< 0.01
Mean AST (SD)/Units per liter	65 (65)	45 (22)	148 (107)	< 0.01
Median FIB-4 (25th, 75th)	1.3 [0.9, 2.1]	1.1 [0.9, 1.5]	3.4 [2.3, 6.6]	< 0.01
Median APRI (25th, 75th)	0.6 [0.4, 1.0]	0.5 [0.3, 0.8]	2.4 [1.5, 4.2]	< 0.01
Median Fibroscan (25th, 75th); *N* = 33 (9 determined to have advanced fibrosis/cirrhosis)	6.4 [5.3, 8.4]	6.2 [5.0, 7.2]	8.0 [7.6, 13.6]	< 0.01
Mean Log10 HVL (SD) /copies/mL	4.5 (1.1)	4.5 (1.0)	4.6 (1.3)	0.85

^a^ heavy drinking (> 4 standard drinks in a day (or > 14 standard drinks/week) for men and > 3/ day (or > 7/week) for women)

Data are N(%) unless otherwise specified.

In unadjusted analyses, the proportions of senescent, memory and naïve CD8+ and CD4+ T-cells were similar by liver fibrosis category (Tables 2, 3, 4, and 5; *p* > 0.05). All associations remained non-significant after adjustment for possible confounders (Tables 4 and 5). In adjusted analyses of the two primary outcomes, the proportion of CD8 + CD28-CD57+ T-cells was lower among those with advanced liver fibrosis/cirrhosis, but the difference was not statistically significant (adjusted mean difference [95% confidence interval, CI]: − 0.36 [− 7.35, 6.63]. CD4 + CD28-CD57+ T-cells proportion was similar for the two groups (adjusted ratio of means [95% CI]: 1.00 [0.48, 2.07]). For this and other back transformed T-cell subsets, the ratio of means is interpreted as follows: mean proportion of T-cell subset in the advanced liver fibrosis/cirrhosis group compared to the group without advanced liver fibrosis/cirrhosis.

**Table 2:** Distribution of CD8+ T-cell subsets reflecting immunosenesence by liver fibrosis status among art-naïve PLWHA with recent heavy alcohol use

T-cell subsets as a proportion of CD8+ T-cells	Mean (SD) unless otherwise stated			*p*-value
	Overall	No advanced fibrosis/cirrhosis	Advanced fibrosis/cirrhosis	
CD8 + CD28-CD57+	35.6 (15.7)	35.4 (15.5)	36.1 (16.7)	0.85
CD8+ memory	34.4 (13.8)	34.9 (13.7)	32.3 (14.4)	0.40
CD8+ naive	38.7 (15.0)	39.3 (14.0)	36.4 (18.6)	0.44
CD8 naïve:memory ratio median (25th, 75th)	1.56 (0.99, 2.40)	1.54 (0.95, 2.30)	1.82 (1.17, 3.00)	0.26

**Table 3:** Distribution of CD4+ T-cell subsets reflecting immunosenesence by liver fibrosis status among art-naïve PLWHA with recent heavy alcohol use

T-cell subsets as a proportion of CD4+ T-cells	Mean (SD) unless otherwise stated			*p*-value
	Overall	No advanced fibrosis/cirrhosis	Advanced fibrosis/cirrhosis	
CD4 + CD28-CD57+ (25th, 75th)	2.25 (0.40, 6.40)	2.40 (0.40, 6.10)	1.40 (0.40, 6.60)	0.59
CD4+ memory	54.3 (16.3)	53.7 (15.1)	56.6 (20.7)	0.42
CD4+ naive	38.7 (15.0)	39.3 (14.0)	36.4 (18.6)	0.39
CD4+ naïve:memory ratiomedian (25th, 75th)	0.70 (0.48, 1.07)	0.75 (0.48, 1.13)	0.59 (0.47, 1.06)	0.81

**Table 4:** a) Unadjusted and b)adjusted association between liver fibrosis status and CD8+ T-cell subsets reflecting immunosenesence among art-naïve PLWHA with recent heavy alcohol use

a)	CD8+ CD28- CD57+ (as % of CD8+)		CD8+ Memory (as % of CD8+)		CD8+ Naïve (as % of CD8+)		CD8+ Naïve:Memory ratio	
	Unadjusted Mean Difference (95% CI)	*P*-value	Unadjusted Mean Difference (95% CI)	*P*-value	Unadjusted Mean Difference (95% CI)	*P*-value	Unadjusted Ratio of Means^a^ (95% CI)	*P*-value
No advanced fibrosis/ cirrhosis	1 (referent group)		1 (referent group)		1 (referent group)		1 (referent group)	
Advanced fibrosis/ cirrhosis	0.67 (−6.15, 7.48)	0.85	−2.61 (−8.59, 3.36)	0.39	2.48 (−3.78, 8.73)	0.44	1.24 (0.84, 1.90)	0.25
b)	CD8+ CD28- CD57+ (as %CD8+)		CD8+ Memory (as %CD8+)		CD8+ Naïve (as%CD8+)		CD8+ Naïve:Memory ratio	
	Adjusted Mean Difference (95% CI)	*P*-value	Adjusted Mean Difference (95% CI)	*P*-value	Adjusted Mean Difference (95% CI)	*P*-value	Adjusted Mean Difference (95% CI)	*P*-value
No advanced fibrosis/ cirrhosis	1 (referent group)		1 (referent group)		1 (referent group)		1 (referent group)	
Advanced fibrosis/ cirrhosis (adjusted)	−0.36 (−7.35, 6.63)	0.92	−3.16 (−8.90, 2.57)	0.28	3.07 (−2.97, 9.11)	0.32	1.32 (0.88, 1.97)	0.18
Age/years	−0.07 (− 0.39, 0.53)	0.77	− 0.15 (− 0.53, 0.23)	0.45	0.01 (− 0.39, 0.41)	0.96	1.00 (0.97, 1.03)	0.96
Female sex	−0.53 (−6.93, 5.87)	0.87	−3.12 (− 8.37, 2.13)	0.24	2.76 (−2.76, 8.29)	0.33	1.23 (0.85, 1.78)	0.27
Log_10_(HIV-1 RNA copies/mL)	−0.18 (−2.70, 2.34)	0.89	2.94 (0.87, 5.01)	< 0.01	−2.38 (−4.55, − 0.20)	0.03	0.91 (0.79, 1.05)	0.20
Heavy drinking	−0.15 (−9.45, 9.16)	0.98	−6.68 (−14.32, 0.95)	0.09	8.27 (0.23, 16.30)	0.04	1.47 (0.86, 2.51)	0.16
Injection drug use	3.26 (−3.08, 9.60)	0.31	−1.10 (−6.31, 4.10)	0.68	2.95 (−2.53, 8.42)	0.29	1.26 (0.87, 1.81)	0.22
Current smoking	−0.17 (−8.51, 8.18)	0.97	5.31 (−1.53, 12.16)	0.13	−5.58 (− 12.79, 1.63)	0.13	0.74 (0.46, 1.20)	0.23
Years since HIV diagnosis	−0.16 (− 0.72, 0.41)	0.59	− 0.24 (− 0.70, 0.22)	0.31	0.65 (0.16, 1.14)	< 0.01	1.03 (1.00, 1.07)	0.07
Hepatitis C	6.67 (−2.29, 15.63)	0.14	1.5 (−5.84, 8.86)	0.69	−2.93 (−10.7, 4.81)	0.46	0.89 (0.53, 1.48)	0.65
Hepatitis B	−3.41 (−9.68, 2.86)	0.29	4.72 (−0.42, 9.87)	0.07	−5.55 (−11.0, − 0.13)	0.04	0.67 (0.47, 0.97)	0.03
Shingles	1.70 (−5.02, 8.43)	0.62	3.04 (−2.48, 8.55)	0.28	−1.64 (−7.44, 4.17)	0.58	0.85 (0.58, 1.25)	0.41
Tuberculosis	2.43 (−6.07, 10.93)	0.58	−8.60 (−15.57, − 1.62)	0.02	6.07 (− 1.27, 13.41)	0.11	1.46 (0.89, 2.37)	0.13

^a^Result after back transformation from natural log scale

**Table 5:** a) Unadjusted and b) adjusted association between liver fibrosis status and CD4+ T-cell subsets reflecting immunosenesence among art-naïve PLWHA with recent heavy alcohol use

a)	CD4+ CD28- CD57+ (as % of CD4+)		CD4+ Memory (as % of CD4+)		CD4+ Naïve (as % of CD4+)		CD4+ Naïve:Memory ratio	
	Unadjusted Mean Difference^a^ (95% CI)	*P*-value	Unadjusted Mean Difference (95% CI)	*P*-value	Unadjusted Mean Difference (95% CI)	*P*-value	Unadjusted Mean Difference^a^ (95% CI)	*P*-value
No advanced fibrosis/ cirrhosis	1 (referent group)		1 (referent group)		1 (referent group)		1 (referent group)	
Advanced fibrosis/ cirrhosis	0.81 (0.39, 1.70)	0.58	2.95 (−4.09, 10.0)	0.41	−3.83 (−9.28, 1.61)	0.17	0.95 (0.63, 1.44)	0.81
b)	CD4+ CD28- CD57+ (as % of CD4+)		CD4+ Memory (as % of CD4+)		CD4+ Naïve (as % of CD4+)		CD4+ Naïve:Memory ratio/CD4+	
	Adjusted Mean Difference^a^ (95% CI)	*P*-value	Adjusted Mean Difference (95% CI)	*P*-value	Adjusted Mean Difference (95% CI)	*P*-value	Adjusted Mean Difference^a^ (95% CI)	*P*-value
No advanced fibrosis/ cirrhosis	1 (referent group)		1 (referent group)		1 (referent group)		1 (referent group)	
Advanced fibrosis/ cirrhosis	1.00 (0.48, 2.07)	1.00	2.15 (−4.89, 9.19)	0.55	−3.38 (−8.67, 1.91)	0.21	1.00 (0.66, 1.52)	0.99
Age/years	1.05 (1.00, 1.10)	0.07	0.32 (−0.15, 0.79)	0.18	−0.37 (− 0.79, 0.04)	0.08	0.98 (0.96, 1.01)	0.22
Female sex	0.89 (0.46, 1.73)	0.73	−2.42 (−8.86, 4.02)	0.46	2.97 (− 2.62, 8.57)	0.30	1.14 (0.78, 1.67)	0.49
Log_10_(HIV-1 RNA)	0.91 (0.70, 1.18)	0.49	0.79 (−1.75, 3.33)	0.54	−0.59 (− 2.58, 1.41)	0.56	1.00 (0.86, 1.17)	0.96
Heavy drinking	0.91 (0.34, 2.39)	0.84	−4.87 (−14.23, 4.50)	0.31	5.36 (−2.71, 13.43)	0.19	1.27 (0.73, 2.21)	0.40
Injection drug use	1.09 (0.56, 2.11)	0.80	−3.11 (−9.50, 3.27)	0.34	3.53 (−1.9, 8.96)	0.20	1.16 (0.80, 1.69)	0.43
Current smoking	0.80 (0.34, 1.91)	0.62	1.75 (−6.65, 10.15)	0.68	−2.3 (−9.75, 5.16)	0.55	0.92 (0.56, 1.51)	0.73
Years since HIV diagnosis	0.98 (0.93, 1.04)	0.55	−0.06 (− 0.63, 0.51)	0.83	0.11 (− 0.38, 0.6)	0.66	1.00 (0.97, 1.04)	0.83
Hepatitis C	0.37 (0.14, 0.93)	0.04	−2.57 (−11.59, 6.45)	0.58	3.49 (−4.52, 11.5)	0.39	1.14 (0.67, 1.94)	0.63
Hepatitis B	0.62 (0.32, 1.19)	0.15	5.69 (−0.62, 12.01)	0.08	−5.04 (−10.39, 0.3)	0.06	0.74 (0.51, 1.07)	0.11
Shingles	1.41 (0.70, 2.84)	0.34	6.13 (−0.63, 12.90)	0.08	−5.51 (−11.32, 0.31)	0.06	0.71 (0.47, 1.05)	0.09
Tuberculosis	0.93 (0.38, 2.24)	0.86	1.45 (−7.10, 10.01)	0.74	−3.64 (−10.84, 3.56)	0.32	0.82 (0.50, 1.37)	0.46

^a^Represents the ratio of means after back transformation from natural log scale

In secondary analyses, the ratio of naïve:memory CD8+ T-cells was higher for those with advanced liver fibrosis/cirrhosis (adjusted ratio of means [95% CI]: 1.32 [0.88, 1.97]). The ratio of naïve:memory CD4+ T-cells was similar for the two groups (adjusted ratio of means [95% CI]: 1.00 [0.66, 1.52]). Confirmatory analysis with median regression for CD4 + CD28-CD57+ T-cells yielded results consistent with the primary analyses.

In exploratory analyses of FIB-4 as a continuous variable, a piecewise linear model was fit for the outcome CD8 + CD28-CD57+ T-cells. Although the observed slope for FIB-4 was positive at lower levels of FIB-4 and negative for higher levels of FIB-4, neither reached statistical significance (Table 6). For the outcome CD4 + CD28-CD57+ T-cells, a linear model was fit based on the GAM, and no significant association was observed for FIB-4.

**Table 6:** Association between liver fibrosis status (continuous measure) and a) CD8+ T-cell b) CD4+ T-cell subsets reflecting immunosenesence among art-naïve PLWHA with recent heavy alcohol use

a)	CD8+ CD28- CD57+ (as % of CD8+)	
	Adjusted Mean Difference (95% CI)	*P*-value
Log(FIB-4) ≤ 1	6.2 (− 0.70, 13.18)	0.08
Log(FIB-4) > 1	−5.54 (− 13.6, 2.47)	0.17
Age/years	−0.06 (− 0.58, 0.45)	0.80
Female sex	−0.83 (−7.68, 6.01)	0.81
Log_10_(HIV-1 RNA)	−0.45 (− 3.15, 2.25)	0.74
Heavy drinking	1.24 (−8.99, 11.47)	0.81
Injection drug use	3.54 (−3.12, 10.20)	0.30
Current smoking	−0.55 (−9.53, 8.43)	0.90
Years since HIV diagnosis	−0.08 (− 0.68, 0.52)	0.79
Hepatitis C	4.32 (−5.33, 13.97)	0.38
Hepatitis B	−3.69 (− 10.34, 2.97)	0.27
Shingles	0.32 (−6.82, 7.46)	0.93
Tuberculosis	3.11 (−5.82, 12.04)	0.49
b)	CD4+ CD28- CD57+ (as% of CD4+)	
	Adjusted Mean Difference (95% CI)	*P*-value
Log(FIB-4)	0.99 (0.64, 1.54)	0.97
Age/years	1.05 (0.99, 1.10)	0.09
Female sex	0.83 (0.41, 1.71)	0.62
Log_10_(HIV-1 RNA)	0.90 (0.68, 1.20)	0.48
Heavy drinking	1.07 (0.37, 3.14)	0.90
Injection drug use	1.09 (0.54, 2.19)	0.81
Current smoking	0.89 (0.35, 2.27)	0.80
Years since HIV diagnosis	0.99 (0.93, 1.05)	0.68
Hepatitis C	0.34 (0.13, 0.93)	0.04
Hepatitis B	0.62 (0.31, 1.24)	0.18
Shingles	1.34 (0.63, 2.81)	0.44
Tuberculosis	0.92 (0.36, 2.36)	0.87

## Discussion

In this exploratory study of ART-naïve PLWHA with heavy drinking, those with advanced liver fibrosis/cirrhosis appeared to have lower prevalence of CD8+ T-cell phenotypes suggestive of immunosenescence (i.e. lower proportions CD8 + CD28-CD57+, higher naïve:memory CD8+ T-cell ratio). However, these differences did not reach statistical significance.

To our knowledge, no published studies have assessed these T-cell subpopulations by liver fibrosis status among HIV infected people. A prior study described similar memory (CD45RO+) T-cell proportions among HIV uninfected patients with cirrhosis compared to healthy controls, which is consistent with our results. Markers of immunosenescence in that study were increased in cirrhosis patients compared to healthy controls (i.e., higher proportion of CD8 + CD45RO + CD57+ T-cells) and in cirrhotic patients with ascites compared to healthy controls (i.e., lower proportion of CD28+ T-cells) [25]. Others have reported evidence of immunosenescence (i.e., lower CD8 + CD28+ T-cell proportions) among HIV uninfected people with cirrhosis who consumed > 90 g/day of ethanol in prior 5 years compared to healthy controls consuming 10-15 g/day ethanol [26]. These findings may differ from ours because: 1) our study was conducted among PLWHA; 2) we investigated different subsets of T-cells representing immunosenescence; and 3) we had few people with cirrhosis and did not have data about ascites.

HIV and viral hepatitis have each been shown to be associated with markers of cellular immunosenescence. Since all participants in the current study were ART-naïve PLWHA and most were hepatitis C co-infected, it is plausible that the effects of HIV and hepatitis C infection on the T-cell subsets we assessed may be masking a potential effect of liver fibrosis on these cell populations. Evidence for this is provided by the associations we observed linking HIV-1 RNA and hepatitis C co-infection with CD8+ and CD4+ T-cell subsets respectively. Future studies should repeat these analyses among HIV infected people with suppressed viremia.

Another potential reason these results do not confirm our hypothesis involves the regenerative capability of the liver. Many of the participants in the prior studies where differences in T-cell subsets were observed had biopsy confirmed cirrhosis and/or ascites. It is possible that a much greater degree and chronicity of liver fibrosis or cirrhosis than was present in our cohort, is required before differences demonstrating senescence in T-cell subsets will be observed. If this were to be true, it would suggest that subclinical measures of liver fibrosis as used in this study may not be able to identify if the liver is losing its ability to regulate immune activation and maintain immune tolerance. Alternatively, it could also suggest that a longer duration of liver fibrosis may be required before subclinical measures of liver fibrosis can provide information about senescence.

Our exploratory analyses suggest the importance of considering non-linear associations between liver disease and T-cell subsets consistent with immunosenescence. While FIB-4 of 3.25 is an important clinical threshold for advanced fibrosis/cirrhosis, there is ambiguity in interpreting FIB-4 between 1.45 and 3.25. Our analyses suggest that the relationship between FIB-4 and CD8+ T-cells with senescent characteristics could potentially vary within this indeterminate range. Future studies linking liver disease and T-cell dysfunction should examine this further and account for the possibility of non-linear associations.

Limitations to this work that warrant discussion. Currently, more detailed markers of immunosenescence and memory and naïve T-cell phenotypes available than were readily available at the time of our flow cytometry testing between 2013 and 2015. Our analysis only examined T-cell phenotypes as percentages of CD4 or CD8, where an absolute count, which was unavailable, might yield different conclusions. Imaging confirmation of liver fibrosis was only available for a subset of our participants. In this subset however, Fibroscan data suggest we achieved good separation of the liver fibrosis exposure groups we defined. Several of our covariates were self-reported, without information on duration of co-morbid conditions, and subject to reporting bias. However, the several potential confounders (e.g. alcohol, HIV viremia, hepatitis C status) were measured using validated instruments and/or objective laboratory data. This study was likely underpowered to detect the associations of interest and as such should be considered exploratory and hypothesis generating. Post hoc power calculations based on the observed standard deviations in each group indicate we had approximately 80% power to detect a minimum difference in CD8 + CD28-CD57+ T-cell proportions of 10, a substantially larger mean difference than the 0.7 observed in this study. As with all observational studies, there is a possibility of unmeasured confounding.

## Conclusions

We did not detect a significant association between advanced liver fibrosis/cirrhosis and the senescent T-cell phenotypes assessed in this sample of ART-naïve HIV-infected Russians with heavy alcohol consumption.

## Data Availability

The datasets used and/or analyzed during the current study are available from the corresponding author on reasonable request.
